# Influence of gelatin type on physicochemical properties of electrospun nanofibers

**DOI:** 10.1038/s41598-023-42472-9

**Published:** 2023-09-14

**Authors:** Bruna Silva de Farias, Francisca Zuchoski Rizzi, Eduardo Silveira Ribeiro, Patrícia Silva Diaz, Tito Roberto Sant’Anna Cadaval Junior, Guilherme Luiz Dotto, Mohammad Rizwan Khan, Salim Manoharadas, Luiz Antonio de Almeida Pinto, Glaydson Simões dos Reis

**Affiliations:** 1 School of Chemistry and Food, Federal University of Rio Grande (FURG), km 8 Itália Avenue, Rio Grande, RS 96203-900 Brazil; 2 Biotechnology Unit, Technology Development Center, Federal University of Pelotas (UFPEL), Eliseu Maciel, Capão do Leão, 96010-610 Brazil; 3 Research Group on Adsorptive and Catalytic Process Engineering (ENGEPAC), Federal University of Santa Maria, Av. Roraima, 1000-7, Santa Maria, RS 97105-900 Brazil; 4 Department of Chemistry, College of Science, King Saud University, Riyadh, 11451 Saudi Arabia; 5 Department of Botany and Microbiology, College of Science, King Saud University, Riyadh, 11451 Saudi Arabia; 6 Department of Forest Biomaterials and Technology, Biomass Technology Centre, Swedish University of Agricultural Sciences, SE-901 83 Umeå, Sweden

**Keywords:** Materials science, Biomaterials, Nanoscale materials

## Abstract

This study explores the fabrication of nanofibers using different types of gelatins, including bovine, porcine, and fish gelatins. The gelatins exhibited distinct molecular weights and apparent viscosity values, leading to different entanglement behavior and nanofiber production. The electrospinning technique produced nanofibers with diameters from 47 to 274 nm. The electrospinning process induced conformational changes, reducing the overall crystallinity of the gelatin samples. However, porcine gelatin nanofibers exhibited enhanced molecular ordering. These findings highlight the potential of different gelatin types to produce nanofibers with distinct physicochemical properties. Overall, this study sheds light on the relationship between gelatin properties, electrospinning process conditions, and the resulting nanofiber characteristics, providing insights for tailored applications in various fields.

## Introduction

Gelatin is a heterogeneous mixture of polypeptides obtained through controlled hydrolysis of collagen. In industrial processes, gelatin is produced through acid, base, or mixed pre-treatment, followed by thermal treatment^1,2^. Traditionally, bovine and porcine skin and bones have been the primary sources of gelatin production^3,4^. However, fish skin has emerged as a promising and extensively studied alternative^5–7^. The hydrolysis of collagen leads to the cleavage of hydrogen, peptide, and covalent bonds in tropocollagen, forming free α chains, β chains, and γ chains. These chains consist mostly of repeated sequences of the amino acids Gly-X-Y^8–10^. The specific amino acids X and Y vary depending on the collagen source, influencing gelatin's final amino acid sequence^2^. Importantly, gelatin can restore collagen-like triple helix structures at temperatures within the range of 25–35 °C. These unique or aggregated segments act as junction zones, promoting enhanced intra- and intermolecular interactions along the polypeptide chains. However, above this temperature range, the triple helix dissociates, allowing the solvation of gelatin chains^11,12^.

Among the diverse types of gelatin-based biomaterials, gelatin nanofibers offer several advantages due to their high surface area. These advantages include increased digestibility of pharmaceutical/bioactive compounds, adsorption of bioactive compounds at the intestinal mucosa, adsorption of organic compounds at adsorbents, and enhanced solubility of nonpolar bioactive compounds^13–16^. These unique characteristics, combined with gelatin's properties, especially the presence of nonpolar functional groups, allow for effective interaction with hydrophobic bioactive compounds in developing nanocarriers^17,18^.

In the development of gelatin-based nanofibers, the molecular weight of the polypeptide chain plays a significant role in determining the physicochemical properties of the final nanomaterial ^19^. Different molecular weights of gelatin can be obtained depending on the source of raw materials and operational conditions employed. Type B gelatin typically has a molecular weight range of 40–90 kDa^20^, while type A gelatin has a higher tendency to maintain the integrity of the α chain, with a molecular weight of approximately 90–100 kDa^10,18^. The variability in gelatin molecular weight underscores the importance of characterizing gelatin to understand its influence on developing novel gelatin nanofibers. Previous studies have predominantly centered on investigating the influence of solution concentration, solvent type, and operational parameters on gelatin nanofiber formation^21–26^. However, the molecular weight of gelatin, a critical factor with potential far-reaching effects, has not been a focal point of these investigations. To the best of our knowledge, no previous studies have investigated the effect of different types of gelatins and their molecular weights on nanofiber formation. For instance, this study seeks to address this gap by exploring the impact of bovine, porcine, and fish gelatin on the physicochemical properties of nanofibers.

## Material and methods

### Gelatin

Bovine gelatin (type B, gel strength of 225 g Bloom), porcine gelatin (type A, gel strength of 300 g Bloom), and cold-water fish skin (without gel strength) were commercially purchased (Sigma-Aldrich, Brazil). Cannon–Fenske viscometer evaluated gelatin's viscosity-average molecular weight (*MW*) (Schott Geraete, GMBH-D65719, Germany). The intrinsic viscosity (*η*) was calculated by Huggin’s equation, Eq. (1). Then, the molecular weight was estimated using the Mark-Houwink equation, Eq. (2).

1 $$ \frac{{\eta_{SP } }}{c} = (\eta ) + k(\eta )^{2} c $$

2 $$ \left[ \eta \right] = K(MW)^{a} $$

where *η*_*SP*_/*c* is the reduced viscosity (mL g^−1^), *η*_*SP*_ is the specific viscosity which compares the viscosity of the gelatin in solution to that of the solvent (dimensionless), *c* is the gelatin concentration (g mL^−1^), *k* is the Huggins constant (dimensionless), *K* and *a* are constants, which depends on the system solvent-polymer (*K* = 0.16 mL g^−1^ and *a* = 0.82)^27^.

### Development of gelatin nanofibers

Different concentrations of gelatin (20–35%, w v^−1^) were dissolved in a 30% (v v^−1^) acetic acid solution for 2 h under stirring (300 rpm) (Fisatom, 752, Brazil) at 50 °C. The gelatin nanofibers were produced by electrospinning technique (Instor, Brazil). Based on preliminary tests, the solutions were electrospun using a metallic capillary with a diameter of 0.7 mm, a capillary-to-collector distance of 7 cm, an applied voltage of 25 kV, and a flow rate of 1.2 mL h^−1^. The nanofiber synthesis occurred at a temperature of 25 ± 1 °C and relative humidity of 40 ± 1%.

### Assessment of morphology in gelatin nanofibers

The diameter and surface morphology of bovine, porcine, and fish gelatin nanofibers were examined using a scanning electron microscope (SEM) (JEOL, JSM-6610, Japan) operating at 10 kV. Before imaging, the samples were coated with a 1 nm layer of gold (Denton Vacuum, Desk V, United States)^28^. The average diameter of the nanofibers was determined by randomly measuring the diameter of 50 individual nanofibers in a unique random sample.

### Evaluation of zeta potential

The samples without dilution were subjected to electrophoretic light scattering (ELS) analysis (triplicate) at 25 °C to measure the zeta potential of bovine, porcine, and fish gelatin solutions (LitesizerTM 500, Anton Paar, Austria) at 25 °C. The electrolytes present in the emulsions flowed through a capillary channel, leading to different electrophoretic mobilities. These mobilities were determined by analyzing the Doppler frequency shift in scattered light, employing the Helmholtz-Smoluchowski equation (Eq. 4). Zeta potential values (*ζ*) were acquired using the Kalliope™ software program (Anton Paar, Austria).

3 $$ \zeta = \frac{{dU_{str} }}{d\Delta p} \times \frac{{\eta_{e} }}{{\varepsilon \times \varepsilon_{o} }} \times k $$

where *η*_*e*_ is electrolyte viscosity (Pa s^−1^), *ε* is the relative dielectric constant (F m^−1^), *ε*_*o*_ is the vacuum dielectric constant (F m^−1^), *dU*/*dp* is the slope of streaming potential versus differential pressure (V Pa^−1^), *k* is the electric conductivity of solution (S m^−1^).

### Determination of fluid rheology

The rheological properties of bovine (30% w v^−1^), porcine (20% w v^−1^), and fish (35% w v^−1^) gelatin solutions were assessed by measuring their apparent viscosity (*η*_*a*_) using a rheometer (HAAKE, model RS150, United States), equipped with a cone-plate sensor (C60/2°) with a gap set at 0.104 mm. The measurements were conducted at a range of shear rates from 1 to 200 s^−1^ at 25 °C. The experimental data were fitted to the power law model, described by Eq. (4)^29^. To control nanofiber formation, optimize the process, and ensure scalability and reproducibility, the maximum shear rate (*γ*_max_) was estimated considering the power law and tubular geometry according to Eq. (5)^30^.

4 $$ \tau = K_{i} \gamma^{n} $$

where *τ* is the shear stress (Pa), *K*_*i*_ is the consistency index (Pa s^n^), *γ* is the shear rate (s^−1^), and *n* is the flow behavior index (dimensionless).

5 $$ \gamma_{\max } = \left( {\frac{3n + 1}{{4n}}} \right)\frac{4Q}{{\pi R^{3} }} $$

where *γ*_max_ is the maximum shear rate (s^−1^), *n* is the flow behavior index (dimensionless), *Q* is the volumetric flow rate of the gelatin solution (m^3^ s^−1^), and *R* is the radius of the metallic capillary (m).

### Analysis of structural modifications in gelatin nanofibers

The structural changes in the bovine, porcine, and fish gelatin nanofibers were verified by attenuated total reflectance infrared spectroscopy (ATR-FTIR) (Shimadzu, Prestige 21, Japan). The ATR-FTIR analysis was performed at 20 °C, covering a range of 650–4000 cm^−1^^31^.

### Evaluation of crystallinity in gelatin nanofibers

The evaluation of crystalline modifications in the bovine, porcine, and fish gelatin nanofibers was performed using X-ray diffraction (XRD) (Shimadzu, XD3A, Japan). The XRD analysis was conducted at 40 kV and 40 mA, with a diffraction angle 2θ ranging from 5° to 90° in steps of 0.05°. The distance between consecutive layers of atoms, represented as d (Å), was calculated using Bragg’s Law (Eq. 6)^32^.

6 $$ n_{R} \lambda = 2d \sin \theta $$

where *n* is the reflection order (dimensionless), *λ* is X-ray wavelength (1.5418 Å), and *θ* is the angle of incidence (°).

### Determination of thermal properties in gelatin nanofibers

The thermal properties of bovine, porcine, and fish gelatin nanofibers were investigated by differential scanning calorimetry (DSC) (Shimadzu, DSC-60, Japan), as well as thermogravimetric analysis (TGA) (Shimadzu, TGA-50, Japan). DSC analysis was employed to examine the physical transitions and was performed with a nitrogen flow rate of 50 mL min^−1^ (from 25 to 500 °C) and a heating rate of 10 °C min^−1^. TGA analysis, on the other hand, assessed the thermal stability of the samples and was conducted under a nitrogen flow rate of 30 mL min^−1^ (from 25 to 500 °C) at a heating rate of 10 °C min^−1^.

### Statistical analysis

Statistical analysis included the comparison of nanofibers' diameter. Mean differences were determined using Statistica 7.0 software (StatSoft, United States), with significance set at a 95% confidence level (*p* < 0.05). Parameter estimation for the power law was performed using the nonlinear Quasi-Newton method in Statistica 7.0 (StatSoft, United States). Graphical representations were generated using OriginPro 8.5 software (OriginLab, United States).

## Results and discussion

### Characteristics of bovine, porcine, and fish gelatins

The bovine, porcine, and fish gelatins exhibited a viscosity-average molecular weight of 48.8 ± 2.8, 98.4 ± 1.3, and 24.8 ± 1.5 kDa, respectively. The disparity in these values can primarily be attributed to the gelatin extraction processes. The covalent bonds between aldehyde and free amine groups within tropocollagen chains undergo denaturation during gelatin production^33,34^. Additionally, the hydrogen bonds between the α-type chains comprising the collagen triple helix and a portion of the peptide bonds within the α-type chains are hydrolyzed. Consequently, the molar weight of gelatin is contingent upon its composition, including free α-type chains, depolymerized α chains, β-type chains (comprised of two covalently linked α chains), and γ-type chains (comprised of three covalently linked α chains)^9,10,35^. Therefore, lower molecular weight values can be attributed to implementing more rigorous operating conditions (such as pH, temperature, and time) and applying basic pre-treatment, both of which contribute to increased hydrolysis along the collagen chains.

### Morphology of bovine, porcine, and fish gelatin nanofibers

**Figure 1:**
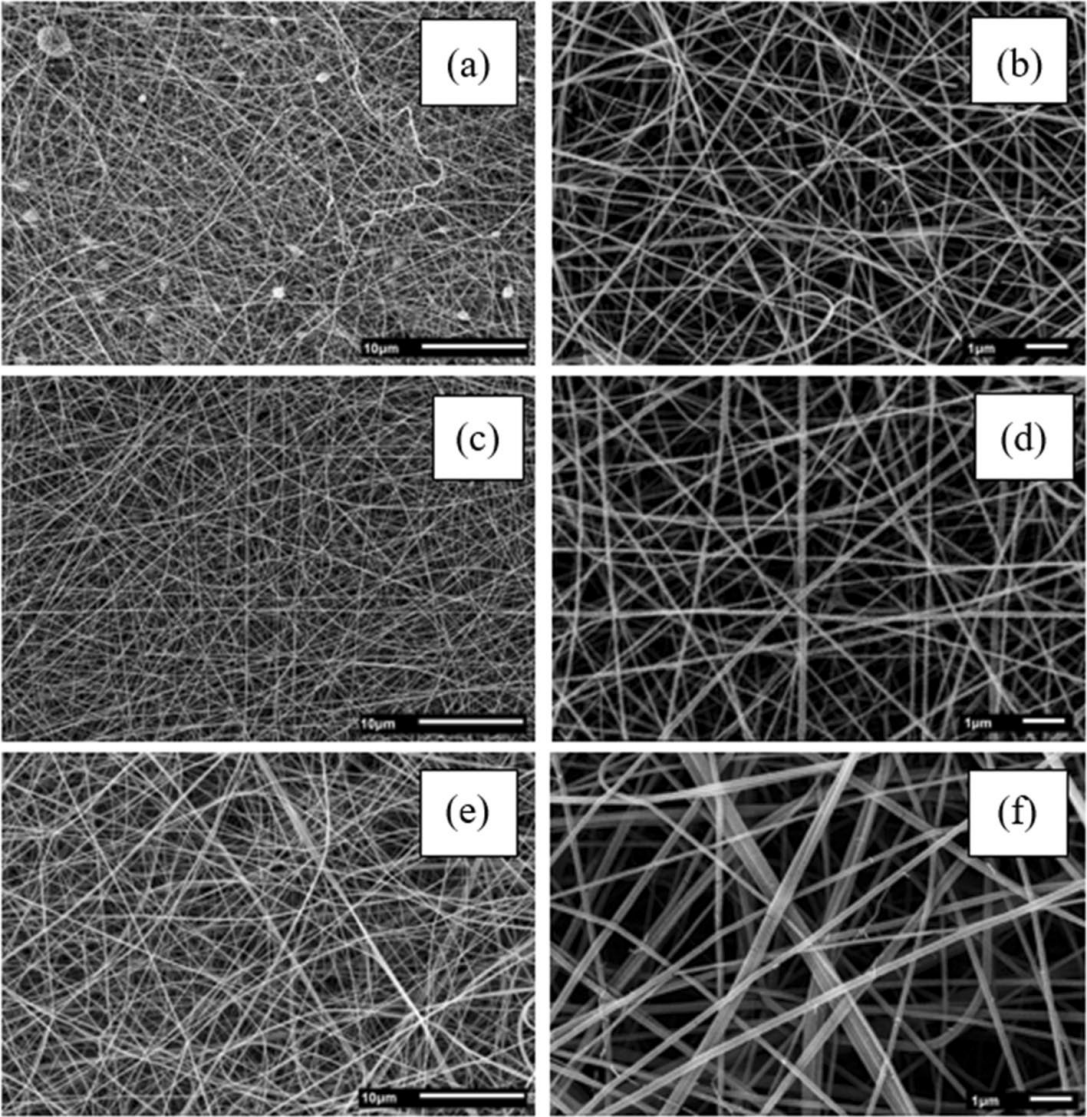
Scanning electron microscope images of bovine gelatin fibers: 20% [(**a**) × 2500; (**b**) × 10,000]; 25% [(**c**) × 2500; (**d**) × 10,000]; 30% [(**e**) × 2500; (**f**) × 10,000].

Figure 1 shows SEM images of bovine gelatin fibers at varying concentrations (20–30% w v^−1^). These images revealed that a concentration of 20% w v^−1^ yielded a combination of droplets and fibers, while higher concentrations (25–30% w v^−1^) resulted in uniformly structured fibers. Moreover, concentrations starting from 20% w v^−1^ represent the minimum threshold for polymer chains to overlap and form fibers. The increase in concentration (25–30% w v^−1^) promotes enhanced interaction and entanglement among the chains, crucial for maintaining a stable jet and preventing the formation of droplet-laden or spherical fibers as observed at 20% w v^−1^ (Fig. 1a). Indeed, Rayleigh instability arises due to opposing forces acting on the jet's surface area. Electrostatic repulsion of charges along the jet increases its surface area. However, if the influence of surface tension surpasses that of viscosity, the jet fragments into droplets to minimize surface area and achieve a lower energy state^19,36^. Concentrations above 30% w v^−1^ impeded fluid flow due to the heightened viscosity, making the fibers' formation unattainable.

**Figure 2:**
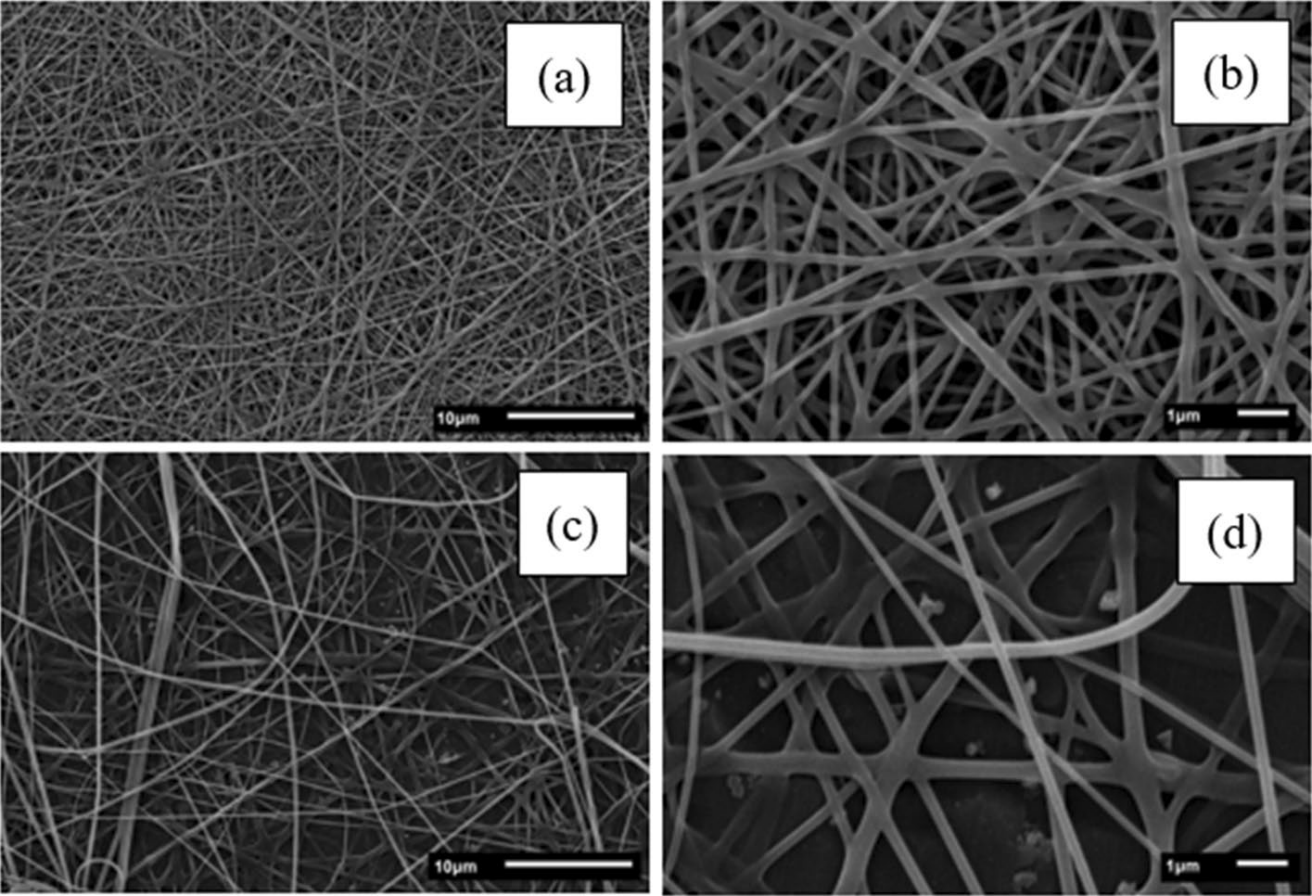
Scanning electron microscope images of porcine gelatin fibers: 20% [(**a**) × 2500; (**b**) × 10,000]; 25% [(**c**) × 2500; (**d**) × 10,000].

Figure 2 displays SEM images of porcine gelatin fibers at different concentrations (20–25% w v^−1^). Notably, a concentration of 20% w v^−1^exclusively yielded fibers, unlike the results observed with bovine gelatin. This distinction can be attributed to the higher molar weight of porcine gelatin. Consequently, lower concentrations enable the necessary entanglement of polymer chains for fiber formation. Figure 2 highlights the presence of branched fibers, potentially linked to secondary jet formation originating from the primary jet. Elongation and solvent evaporation can alter the shape and charge distribution along the jet. Consequently, an imbalance between electric forces and surface tension destabilizes the jet, leading to the emergence of secondary jets that decrease charge density per unit surface area^36,37^.

**Figure 3:**
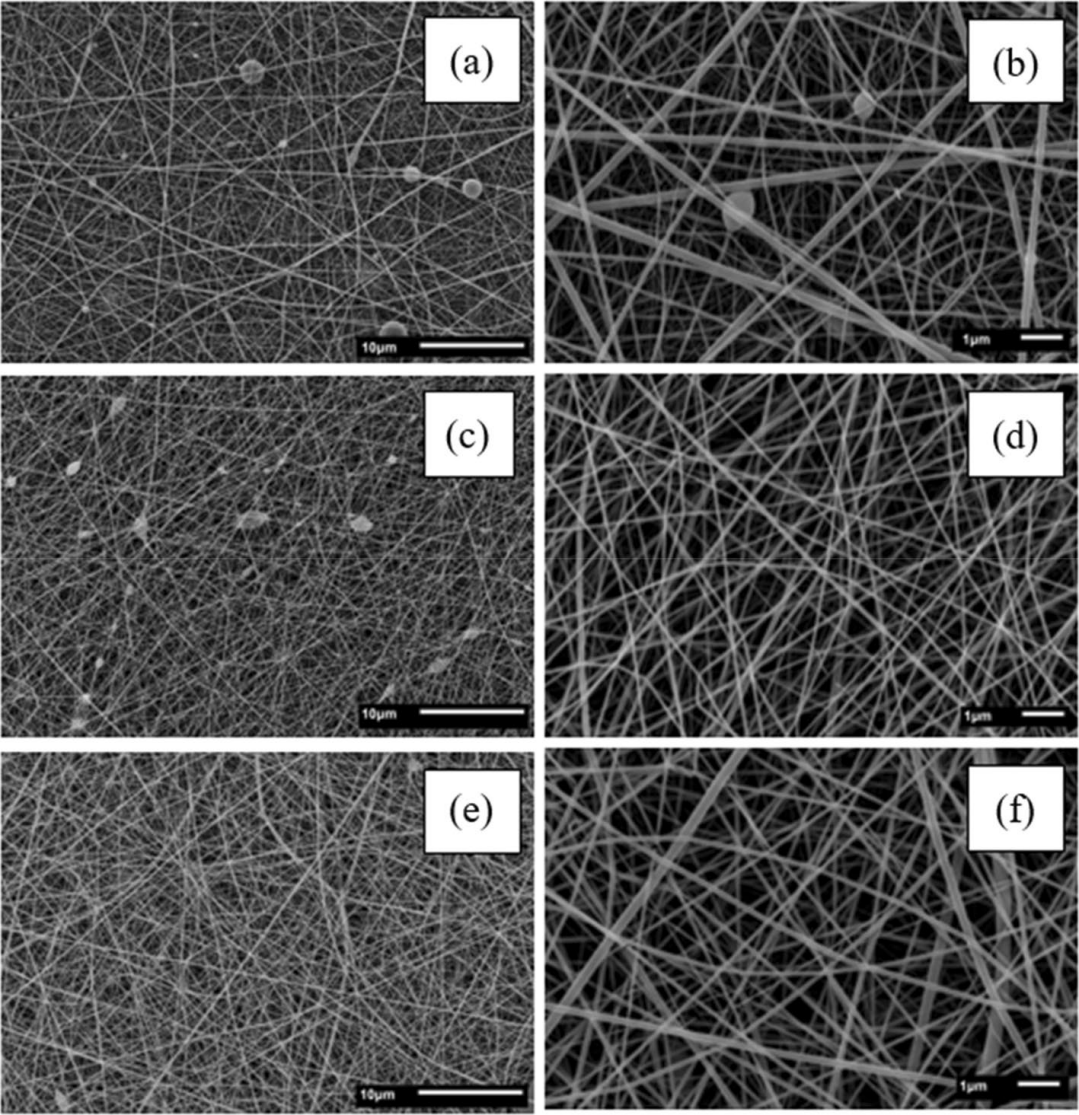
Scanning electron microscope images of fish gelatin fibers: 25% [(**a**) × 2500; (**b**) × 10,000]; 30% [(**c**) × 2500; (**d**) × 10,000]; 35% [(**e**) × 2500; (**f**) × 10,000].

Figure 3 exhibits SEM images of fish gelatin fibers at different concentrations (25–35% w v^−1^). In Fig. 3, the minimum concentration of fish gelatin required to produce fibers was 25% w v^−1^, higher than the minimum concentration required for bovine and porcine gelatin. This difference can be directly attributed to fish gelatin's lower molar weight than the other types. Consequently, a higher concentration of fish gelatin is necessary to achieve the required entanglement of polymer chains for fiber formation. The effect of the reduced molecular weight of fish gelatin also contributed to an increase in the minimum concentration (35% w v^−1^) necessary for forming a stable polymeric jet and, subsequently, producing droplet-free fibers.

Table 1 shows the average diameters of gelatin fibers obtained via the electrospinning process using bovine, porcine, and fish gelatin. All samples, under the investigated operational conditions, yielded nanoscale fibers. Notably, an increase in polymer concentration resulted in a significant (*p* < 0.05) increase in the average diameter of nanofibers across various concentrations and gelatin types^19,38^. This behavior can be attributed to the heightened entanglement of polymer chains, which influences both the jet's stability and the fibers' final diameter^19^. Furthermore, for the same solvent, an augmented concentration curtails the mobility between polymer chains, thereby limiting the stretching of the jet and leading to the formation of larger-diameter fibers^39,40^. Moreover, under the conditions of 25% (w v^−1^), it was observed that all types of gelatin produced nanofibers. Porcine gelatin exhibited the largest nanofibers diameters, followed by bovine and fish gelatins. This difference in nanofibers diameters can be attributed to their distinct molecular weights, as previously mentioned.

**Table 1:** Diameter of bovine, porcine, and fish gelatin nanofibers.

Concentration (%, w v^−1^)	Bovine gelatin	Diameter (nm)	
		Porcine gelatin	Fish gelatin
20	47 ± 14^a^	168 ± 60^a^	–
25	89 ± 25^b^	274 ± 87^b^	68 ± 17^a^
30	110 ± 24^b^	–	76 ± 22^a^
35	–	–	109 ± 48^b^

Mean value ± standard deviation (n = 3). Small letters with different superscripts in the same column differ significantly (p < 0.05).

The samples that yielded the smallest average diameter and were free of droplets were obtained at concentrations of 25% w v^−1^and 30% w v^-−1^ for bovine gelatin, 20% w v^−1^ for porcine gelatin, and 35% w v^−1^ for fish gelatin. The bovine gelatin samples at 25% w v^−1^and 30% w v^−1^exhibited production rates of 0.1 and 0.5 g h^−1^, respectively. The porcine gelatin sample at 20% w v^−1^ had a production rate of 0.1 g h^−1^, while the fish gelatin sample at 35% w v^−1^ had a production rate of 0.3 g h^−1^. It should be mentioned that these values surpass the average nanofiber production (0.01–0.1 g h^−1^) achieved through the traditional electrospinning process with a single capillary^41,42^. Considering the smaller average diameter, absence of droplets, and higher production rate, the samples with 30% w v^−1^ bovine gelatin, 20% w v^−1^ porcine gelatin, and 35% w v^−1^ fish gelatin were selected for subsequent analyses of structural, crystallinity, and thermal properties.

### Zeta potential of bovine, porcine, and fish gelatin solutions

Table 2 presents the electrophoretic and zeta potential values for bovine, porcine, and fish gelatin solutions. The porcine gelatin solution exhibited the highest electrophoretic and zeta potential values, followed by the bovine and fish gelatin solutions. The acid and alkaline pre-treatment conditions result in commercially available gelatin known as type-A gelatin (isoelectric point: 8–9) and type-B gelatin (isoelectric point: 4–5), respectively^43,44^. These differences can be mainly attributed to the partial deamination of glutamine and asparagine to glutamic acid and aspartic acid during the alkaline pre-treatment of gelatin^45^. Therefore, the enhanced electrophoretic and zeta potential values in porcine gelatin solution could be associated with increased amino acids with positively charged side chains, such as arginine, histidine, and lysine, through gelatin structure.

**Table 2:** Characterization of bovine, porcine, and fish gelatin solutions by electrophoretic light scattering (ELS).

Gelatin	Zeta potential (mv)	Electrophoretic mobility (μm s^−1^ V^−1^ cm)
Bovine	9.0 ± 0.5	0.71 ± 0.01
Porcine	16.1 ± 0.6	1.25 ± 0.05
Fish	4.6 ± 0.3	0.36 ± 0.03

Mean value ± standard deviation (n = 3).

### Fluid rheology of bovine, porcine, and fish gelatin

Table 3 presents the fluid rheology data for bovine, porcine, and fish gelatin solutions. The apparent viscosity values obtained across the shear rate range studied (1–200 s^−1^) revealed that the porcine gelatin solution exhibited the highest viscosity, followed by bovine and fish gelatin solutions. Despite fish gelatin having the highest concentration (35% w v^−1^), followed by bovine (30% w v^−1^) and porcine gelatins (20% w v^−1^), the molecular weight of the gelatins appeared to have a pronounced effect on their viscosity.

**Table 3:** Fluid rheology of bovine, porcine, and fish gelatin solutions.

Shear rate (s^–1^)	Apparent viscosity (mPa s^–1^)		
	Bovine	Porcine	Fish
1.5	1050 ± 7	1550 ± 6	306 ± 4
25	970 ± 6	1080 ± 9	256 ± 3
50	947 ± 4	1060 ± 5	248 ± 5
100	937 ± 5	1040 ± 7	246 ± 4
200	920 ± 7	991 ± 8	246 ± 4

Mean value ± standard deviation (n = 3).

The results suggest that porcine gelatin may possess greater structural integrity, particularly concerning β-type and γ-type chains. The presence of these chains introduces additional physical entanglements and constraints that impede the flow of the polymer, resulting in higher apparent viscosity^19,35,46^. On the other hand, fish gelatin exhibited reduced apparent viscosity values, indicating a higher proportion of free α-type chains and depolymerized α chains^47,48^. Furthermore, the higher positive charge observed in porcine gelatin, followed by bovine and fish gelatins, as indicated by the electrophoretic and zeta potential values, increases electrostatic repulsion. This electrostatic repulsion expands the gelatin chains in the solution, consequently increasing the overall size and volume occupied by the gelatin chains^49–51^. These results suggest that a higher concentration of fish gelatin is required to achieve the necessary chain entanglement to produce nanofibers, as previously discussed.

The power law parameters for bovine, porcine, and fish gelatin solutions are presented in Table 4. The porcine gelatin solution exhibited a higher consistency index (*K*_*i*_) than the other types, consistent with its higher apparent viscosity values. Additionally, the flow behavior index (*n*) was approximately 1 for all samples, indicating the Newtonian behavior of the fluids^29^. This behavior is reflected in Table 3, where slight changes in apparent viscosity values were observed over the shear rate range. Moreover, the maximum shear rate calculated at a flow rate of 1.2 mL h^−1^ for bovine, porcine, and fish gelatin solutions was 1.23, 1.24, and 1.22 s^−1^, respectively.

**Table 4:** Power law parameters for bovine, porcine and fish gelatin solutions.

Gelatin	*N*	*K*_*i*_ (Pa s^n^)	*R*^2^	*RMSE*
Bovine	0.97 ± 0.01	1.10 ± 0.02	0.99 ± 0.01	0.38 ± 0.02
Porcine	0.95 ± 0.01	1.33 ± 0.01	0.99 ± 0.01	0.99 ± 0.06
Fish	1.02 ± 0.01	0.22 ± 0.02	0.99 ± 0.01	0.16 ± 0.01

Mean value ± standard deviation (n = 3).

*n* flow behavior index, *K*_*i*_ consistency index, *R*^*2*^ coefficient of determination, *RMSE* root mean squared error.

Decreasing the feed rate reduces the fluid supply to the syringe system, resulting in a lower shear rate as there is less movement between adjacent fluid layers^52^. These findings hold significance as viscous forces exert a stronger influence with decreasing feed rate in the electrospinning process. Considering these results, it can be argued that the importance of feed rate in electrospinning extends beyond jet velocity, solution transfer rate, and solvent evaporation; it also affects the viscoelastic forces. Lower feeding rates are desirable for solvent evaporation and to surpass the surface tension, avoiding Rayleigh instability^53,54^. On the other hand, increasing the apparent viscosity leads to heightened viscoelastic forces that need to be overcome by the electric forces involved and need to^30,38,55^.

### Structural evaluation of bovine, porcine, and fish gelatin nanofibers

**Figure 4:**
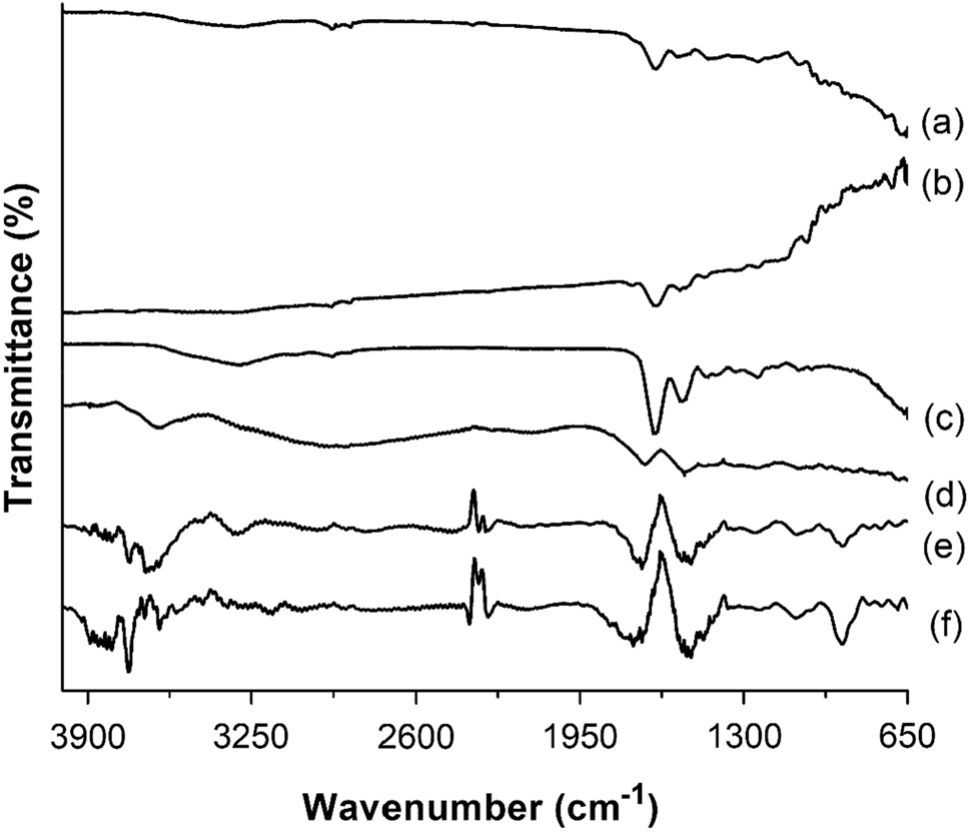
Attenuated total reflectance infrared spectroscopy spectrum: (**a**) bovine gelatin nanofibers; (**b**) porcine gelatin nanofibers; (**c**) fish gelatin nanofibers; (**d**) bovine gelatin powder; (**e**) porcine gelatin powder; (**f**) fish gelatin powder.

Figure 4 depicts gelatin samples’ vibrational spectra in powder and nanofiber forms.

In the spectra of the powder gelatin samples (Fig. 4d, e, f), distinctive bands were observed at specific wavenumbers. At 3625 cm^−1^, the band corresponds to the stretching of NH bonding and hydrogen bonding of amide A. The band at 1750 cm^−1^ represents the stretching of C=O and hydrogen bonding coupled with the COO of amide I. Furthermore, the band at 1518 cm^−1^ indicates the vibration of N–H groups and the stretching of C–N groups of amides II^56,57^. In the vibrational spectra of the gelatin nanofiber samples (Fig. 4a, b, c), alterations in the intensity of the bands were observed. These changes suggest that the electrospinning process could have induced conformational alterations within the structure of the gelatin nanofibers.

### Crystallinity evaluation of bovine, porcine, and fish gelatin nanofibers

**Figure 5:**
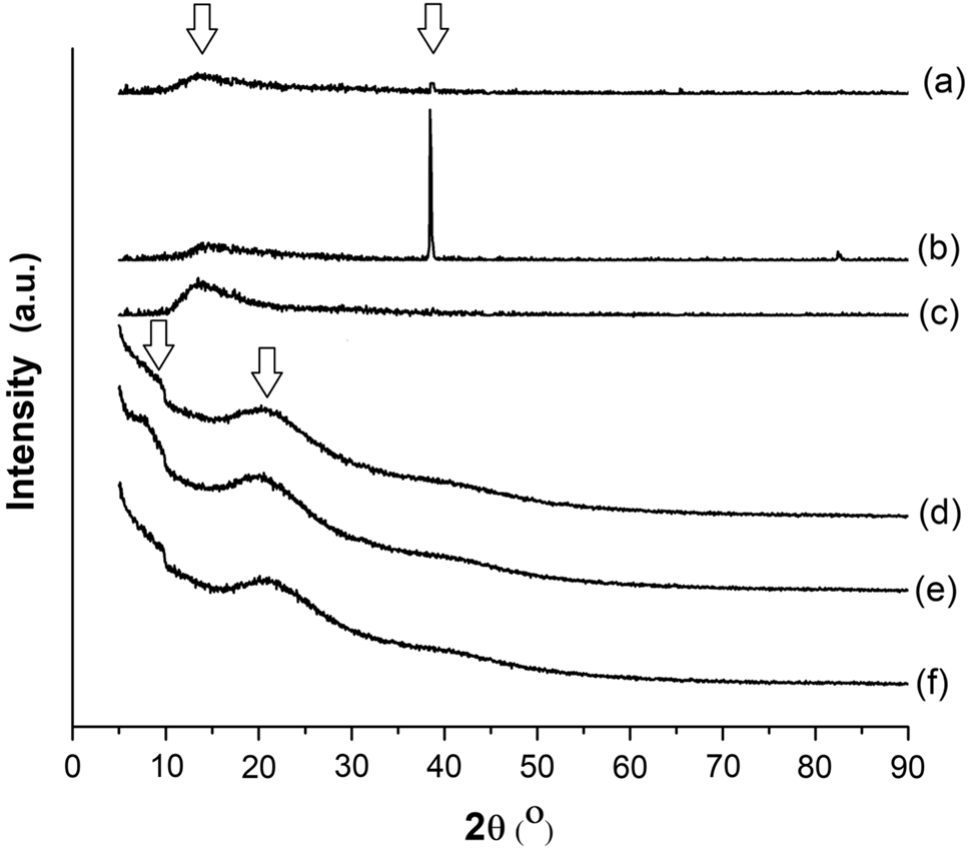
X-ray diffractograms: (**a**) bovine gelatin nanofibers; (**b**) porcine gelatin nanofibers; (**c**) fish gelatin nanofibers; (**d**) bovine gelatin powder; (**e**) porcine gelatin powder; (**f**) fish gelatin powder.

Figure 5 shows the X-ray diffraction patterns of gelatin in both powder and nanofiber forms. In Fig. 5d, e, f, the X-ray diffraction patterns of the gelatin powder samples revealed the presence of two distinct crystalline regions derived from the collagen structure. The first region corresponds to the diameter of the gelatin triple helix, identified at an angle of incidence of 9.0° with a distance of 9.88 Å (first arrow). The second region represents the inter-residue distance within the junction zone of the triple helix, observed at an angle of incidence of 20.0° with a distance of 4.51 Å (second arrow)^11,58,59^. However, changes in the crystallinity of the gelatin samples were observed after the electrospinning process.

Figure 5a, b, c showed a reduction in intensity and alteration of the second crystalline region at an angle of incidence of 14.0° with a distance of 6.37 Å (first arrow). In contrast, the first crystalline region was absent. These findings suggest that the electrospinning process may have disrupted the remaining helical structure of the gelatin, leading to modifications in the junction zones responsible for maintaining the triple helix and ultimately reducing the overall crystallinity of the gelatin samples. Notably, in the nanofiber sample of porcine gelatin (Fig. 5b), a new reflection angle (38.6°) with a distance of 2.47 Å could be observed (second arrow). This result suggests that the electrospinning process induced a new conformational state with enhanced molecular ordering in the nanofibers of porcine gelatin. These observations provide valuable insights into the structural changes and modifications induced by the electrospinning process in gelatin samples.

### Thermal properties of bovine, porcine, and fish gelatin nanofibers

**Figure 6:**
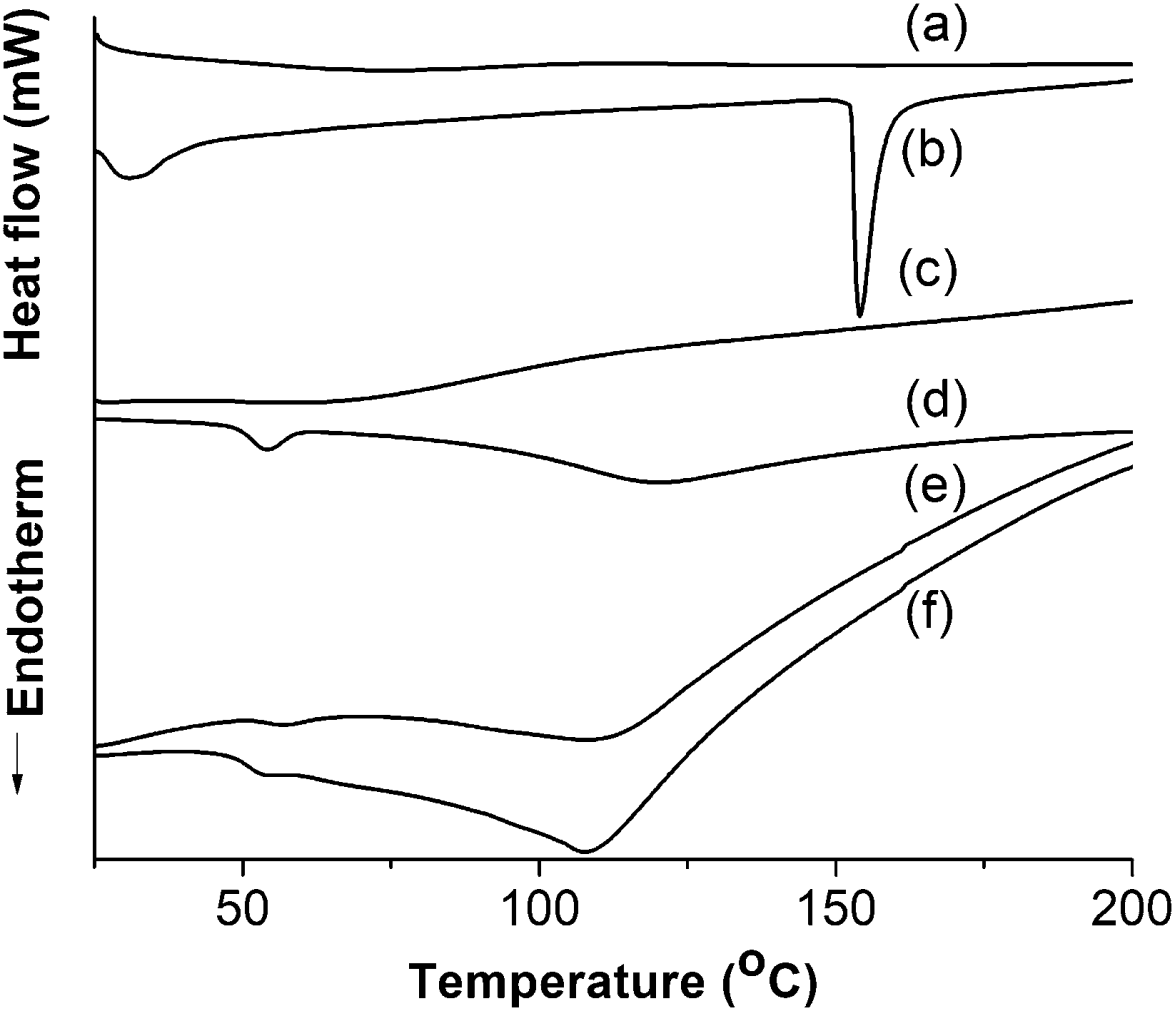
Differential scanning calorimetry curves: (**a**) fish gelatin nanofibers; (**b**) porcine gelatin nanofibers; (**c**) bovine gelatin nanofibers; (**d**) fish gelatin powder; (**e**) porcine gelatin powder; (**f**) bovine gelatin powder.

**Figure 7:**
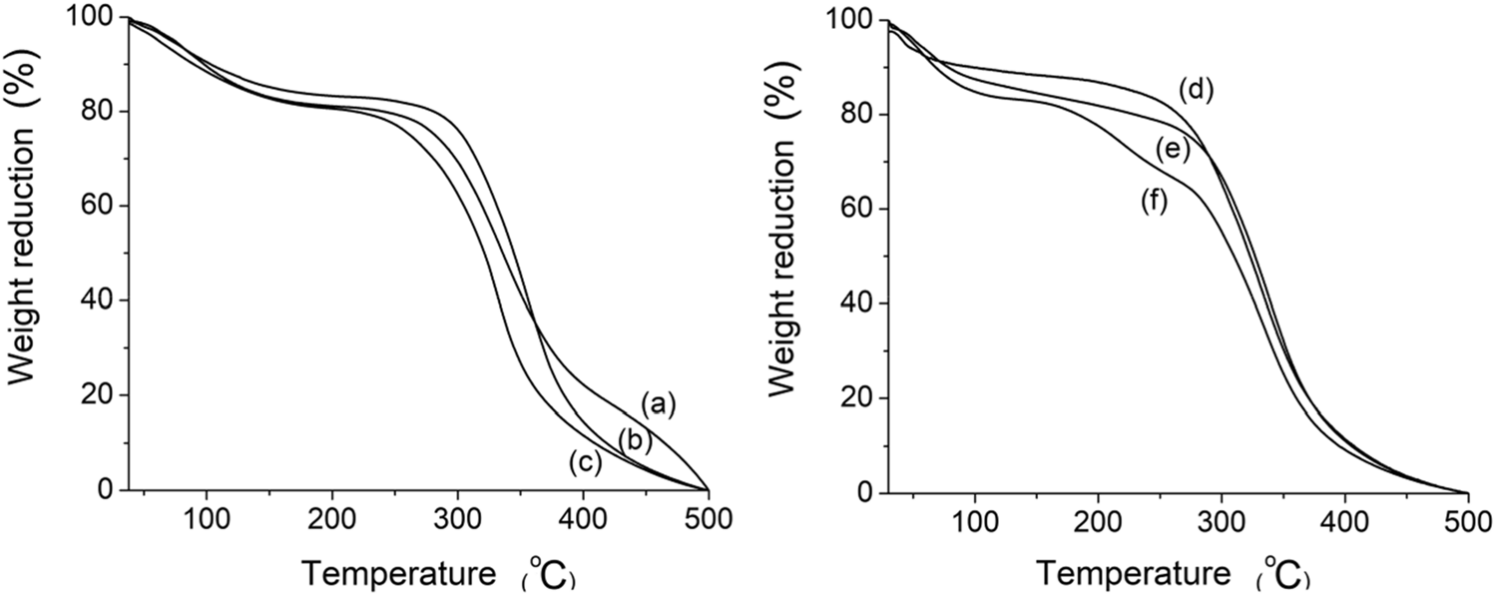
Thermogravimetric analysis curves: (**a**) bovine gelatin powder; (**b**) porcine gelatin powder; (**c**) fish gelatin powder; (**d**) fish gelatin nanofibers; (**e**) bovine gelatin nanofibers; (**f**) porcine gelatin nanofibers.

Figures 6 and 7 present the thermal profiles of gelatin samples (bovine, porcine, and fish) in powder and nanofiber forms, as depicted by the DSC and TGA curves, respectively. The DSC curves of the powder samples (Fig. 6d, e, f) exhibited an endothermic peak around 54 °C, which suggests the evaporation of adsorbed water. This observation is supported by the reduction in weight percentage in the corresponding TGA curves (Fig. 7a, b, c) within the same temperature range, with the maximum evaporation temperature (Te_max_) occurring at 60 °C according to the first derivative of the TGA curves.

The enthalpy of vaporization (∆H_v_) values for bovine gelatin powder, porcine gelatin powder, and fish gelatin powder were determined as 1.1, 2.0, and 20.5 J g^−1^, respectively. The disparity in these values may be attributed to the increased interaction between water and the functional groups of fish gelatin. This trend could result from a higher proportion of smaller-sized polypeptide chains in fish gelatin, such as free α chains and depolymerized α chains, which provide a larger contact surface and greater availability of side chains for interaction with water molecules.

Comparison between the DSC curves of gelatin powder samples (Fig. 6d, e, f) and gelatin nanofiber samples (Fig. 6a, b, c) revealed variations in the exothermic peak associated with water evaporation in fish gelatin nanofibers and porcine gelatin nanofibers. The nanofiber sample from fish gelatin (Fig. 6a) did not exhibit the peak corresponding to the evaporation of adsorbed water, likely due to its lower water content. This result is evident from the lower percentage reduction in weight (~ 6%) in the TGA curve (Fig. 7d) within the temperature range of 0–100 °C, compared to other nanofiber and powder gelatin samples (~ 10–12%) (Fig. 7). However, the nanofiber sample from porcine gelatin (Fig. 6b) displayed changes in the endothermic peak related to water evaporation temperature (31 °C) and ∆H_v_ (69.0 J g^−1^), with Te_max_ occurring at two temperatures (58 and 218 °C). These observations may be attributed to conformational changes in the structure of porcine gelatin during the electrospinning process.

Gelatin exhibits two well-established physical transitions. The first is a second-order transition known as the glass transition temperature (T_g_), while the second is a first-order endothermic transition called melting^60,61^. The glass transition corresponds to the shift from a glassy state, where polymeric chains lack sufficient internal energy for mobility, to a higher energy state, allowing chains in the amorphous region to become flexible and mobile. However, the glass transition was not observed in the gelatin powder samples (Fig. 6d, e, f). Furthermore, an additional endothermic peak was identified in Fig. 6d, e, f, which corresponds to the melting temperature (T_M_) of gelatin. Similar to the glass transition, this peak comprehends the transition from an ordered molecular state to a more disordered state in the crystalline regions of gelatin.

The melting enthalpy (∆H_M_) values for bovine gelatin powder, porcine gelatin powder, and fish gelatin powder were found to be 124.2 J g^−1^ (108 °C), 169.8 J g^−1^ (110 °C), and 266.7 J g^−1^ (118 °C), respectively. As mentioned earlier, the higher ∆H_M_ value in fish gelatin powder compared to bovine gelatin powder could be attributed to the increased interaction between water molecules and functional groups. The adsorbed water molecules could enhance intermolecular interactions and produce a more ordered structure within the fish gelatin^61^. On the other hand, the higher ∆H_M_ value in porcine gelatin powder compared to bovine gelatin powder may be attributed to the preparation process. The acid pre-treatment step can lead to less hydrolysis along the collagen chain, thereby preserving the integrity of the crystalline regions related to β and γ chains.

Comparison between the DSC curves of gelatin samples in powder and nanofiber forms revealed changes in the physical transitions. Figure 6c only displayed a change in the baseline heat flow (mW), which is associated with the glass transition of the amorphous region in bovine gelatin nanofibers (63 °C). Additionally, the nanofiber sample from fish gelatin exhibited T_M_ and ∆H_M_ values of 75 °C and 78.6 J g^−1^, respectively. In comparison, the nanofiber sample from porcine gelatin exhibited T_M_ and ∆H_M_ values of 154 °C and 189.5 J g^−1^. These findings indicate an increase in the amorphous nature of bovine and fish gelatin, whereas an increase in crystallinity was observed in porcine gelatin, aligning with the previously discussed results.

In the nanofiber sample from porcine gelatin, a higher amount of residual solvent may act as an anti-plasticizer, leading to interactions between water molecules and gelatin functional groups. This interaction could intensify intermolecular associations and limit the mobility of smaller chains within the amorphous region of the biopolymer^62,63^. This trend is evident from the previously mentioned increase in ∆H_v_ (69.0 J g^−1^) and the reduction in mass percentage in the TGA curves (Fig. 7f) within two temperature ranges before biopolymer degradation, 25–100 °C (Te_max_ = 58 °C) and 170–270 °C (Te_max_ = 218 °C). The second water evaporation range may be associated with increased water adsorption at the active sites of gelatin. The initial temperature of degradation (T_ID_) for the polymeric chains of bovine gelatin powder, porcine gelatin powder, and fish gelatin powder (Fig. 7a, b, c) occurred at temperatures of 266, 290, and 247 °C, respectively. For gelatins in nanofiber form (bovine, porcine, and fish), the T_ID_ values were measured at 269, 287, and 252 °C, respectively. These results indicate that the electrospinning process did not affect the thermal stability of the nanofibers.

## Conclusion

This study focused on developing nanofibers using different gelatins, namely bovine, porcine, and fish gelatins. The gelatins exhibited distinct molecular weight values: 48.8 ± 2.8, 98.4 ± 1.3, and 24.8 ± 1.5 kDa, respectively. Successful nanofiber formation was achieved for all gelatins, with fiber diameters from 47 to 274 nm. The variations in molecular weight and apparent viscosity among the gelatins indicated that porcine gelatin required lower concentrations to achieve the necessary entanglement for nanofiber production, followed by bovine and fish gelatins. The differences in molecular weight, apparent viscosity, electrophoretic and zeta potential values suggest that the process conditions not only affect the gelatin's chemical structure, including the presence of free α-type chains, depolymerized α chains, β-type chains, and γ-type chains but also impact the side chains of amino acids.

Moreover, the electrospinning process may induce conformational alterations within the gelatin nanofibers, disrupting the remaining helical structure and modifying the junction zones responsible for maintaining the triple helix. This behavior ultimately results in reduced overall crystallinity of the gelatin samples. However, the electrospinning process induced a new conformational state characterized by enhanced molecular ordering in the nanofibers of porcine gelatin. Consequently, this study demonstrates how different gelatins can produce nanofibers with distinct physicochemical properties, influencing their potential applications.

The unique properties of gelatin-based nanofibers make them versatile materials for various industries. Bovine and fish gelatins, with lower crystallinity, increased flexibility and reduced molecular order hold promise for applications requiring higher mass transfer rates, such as oral delivery carriers and membranes for organic compound purification. These nanofibers have higher solubility rate, making them ideal for delivering medications or nutrients in the oral mucosa. Moreover, they can enable higher adsorption capacities, facilitating the separation and purification of valuable compounds in industrial processes. On the other hand, porcine gelatin nanofibers, with increased crystallinity, offer benefits for applications involving prolonged drug/food delivery, food packaging, and biomedical scaffold fabrication. Their dense and structured architecture lends itself to sustained release formulations, ensuring a controlled and extended release of drugs or nutrients over time. In the food packaging industry, these nanofibers can enhance shelf life and protect products from external contaminants. Furthermore, these nanofibers find their place in biomedical engineering, where their structural integrity are critical for the fabrication of tissue scaffolds, promoting cell growth and tissue regeneration. Thus, the selection of gelatin sources during nanofibers production opens up a world of possibilities for tailoring nanofiber properties to specific applications across diverse domains.

## Data Availability

The datasets used and/or analysed during the current study available from the corresponding author on reasonable request.
